# Atomic-Scale Investigation on the Evolution of Tio_2_-Anatase Prepared by a Sonochemical Route and Treated with NaOH

**DOI:** 10.3390/ma13030685

**Published:** 2020-02-04

**Authors:** Berenice Victoria Dimas, Isaías Hernández Pérez, Vicente Garibay Febles, Lucía Díaz Barriga Arceo, Raúl Suárez Parra, Jesús Noé Rivera Olvera, Ricardo Luna Paz, Dulce Viridiana Melo Máximo, Leonardo González Reyes

**Affiliations:** 1 Departamento de Ciencias Básicas, Universidad Autónoma Metropolitana-A, Av. Sn. Pablo No. 180, México D.F. 02200, Mexico; eiby-dyrp@hotmail.com (B.V.D.); lpr@correo.azc.uam.mx (R.L.P.); 2 Instituto Mexicano del Petróleo, Eje Central Lázaro Cárdenas Norte 152 Col. San Bartolo Atepehuacan, México D.F. C.P 07730, Mexico; vgaribay@imp.mx; 3 Departamento de Ingeniería Metalúrgica y Materiales, Instituto Politécnico Nacional, ESIQIE-UPALM, México D.F. 07738, Mexico; luchell@yahoo.com; 4 Instituto de Energías Renovables, IER-UNAM. Priv. Xochicalco S/N, Temixco, Morelos 62580, Mexico; rsp@ier.unam.mx; 5 Tecnológico Nacional de México/ TES Ixtapaluca. TESI, Km. 7 de la carretera Ixtapaluca-Coatepec s/n, Ixtapaluca, Estado de México C.P.56580, Mexico; jnoe.rivera@tesi.edu.mx; 6 Tecnológico de Monterrey, Escuela de Ingeniería y Ciencias, Carretera Lago de Guadalupe km 3.5, Atizapán de Zaragoza C.P. 52926, Mexico; virimelo@tec.mx

**Keywords:** metal oxides, sonochemistry, electron microscopy

## Abstract

To date, the formation mechanisms of TiO_2_, as well as its heterostructures, have not been clarified. Moreover, detailed research on the transition from a tetragonal anatase phase to the monoclinic phase of the TiO_2_(B) phase and their interface structure has been quite limited until now. In the present study, we report on the sonochemical synthesis of TiO_2_-anatase with a crystallite size of 5.2 ± 1.5 nm under different NaOH concentrations via the hydrothermal method. The use of alkaline solution and the effect of the temperature and reaction time on the formation and structural properties of TiO_2_-anatase nanopowders were studied. The effects of NaOH concentration on the formation and transformation of titanate structures are subject to thermal effects that stem from the redistribution of energy in the system. These mechanisms could be attributed to three phenomena: (1) the self-assembly of nanofibers and nanosheets, (2) the Ostwald ripening process, and (3) the self-development of hollow TiO_2_ mesostructures.

## 1. Introduction

Since the first report about water photolysis on titanium dioxide (TiO_2_) electrodes under ultraviolet light irradiation by Fujishinma and Honda in 1972 [1], TiO_2_ nanostructures have been widely studied for optical, electrical, and photochemical applications due to their chemical stability and good performance [2,3,4]. After this important investigation, Marchand et al. reported, almost eight years later in 1980, on the synthesis of a lamellae structure produced by the hydrothermal treatment of TiO_2_ with alkaline solution followed by heat treatment at 500 °C: the synthesized product exhibited a new metastable phase of TiO_2_ that was named the TiO_2_(B) phase due to it showing a similar structure as bronze alloy [5]. This metastable phase of TiO_2_, TiO_2_(B) (*C2/m*), has a less symmetrical monoclinic structure than the common polymorphs of TiO_2_, anatase (tetragonal, *I4/amd*), rutile (tetragonal, *P42/mnm*), and brookite (orthorhombic, Pcab). Certainly, the TiO_2_(B) phase is a less compact structure than the other crystalline structures. The unit volume of TiO_2_(B) is 35.27 Å^3^ as compared to that of 31.12 Å^3^ for rutile, 32.20 Å^3^ for Brookite, and 34.02 Å^3^ for anatase phases [6,7,8]. Furthermore, TiO_2_(B) shows a more open channel in the lattice and the characteristics of pseudocapacitive behavior, resulting in easier Li-ion access to the crystal structure and a faster charge–discharge capability than anatase or rutile phases, for example [9,10,11,12,13,14]. In 1991, Banfield et al. identified and characterized this new metastable phase, TiO_2_(B), which was founded in natural anatase crystals [9]. Finally, in 1998, Kasuga T. et al. reported a new route for the synthesis of TiO_2_ nanotubes through a hydrothermal method: a 5–10 M NaOH aqueous solution and heat treatment at 110 °C for 20 h [10]. However, later studies showed that these structures consisted of hydrated dititanate (H_2_Ti_2_O_5_), trititanate (H_2_Ti_3_O_7_), and H_2_Ti_4_O_9_*·x*H_2_O [15,16,17,18]. These latest reports indicate that the titanate nanostructures combine the properties of conventional titanate (e.g., ion exchange) and TiO_2_ (e.g., photocatalysis) [19]. Therefore, these multirole properties of titanate nanostructures allow them to have such advantages that they can be used as ion-exchangers, adsorbents, and photocatalysts, among other applications. Currently, it is possible to establish that hydrothermal temperature, duration, and the concentration of NaOH are the fundamental stages or parameters that affect the morphology and structures of the products obtained via hydrothermal synthesis. To date, most studies have focused on the effects of hydrothermal temperature and reaction time on the structural properties. However, there are few previous studies reporting the role of particle size on the thermal stability of TiO_2_ nanoparticles under hydrothermal conditions [20,21,22,23,24]. The formation mechanism of TiO_2_ heterostructures has not yet been clarified. Thus, detailed research on the formation of the Ti_n_O_2n-1_ phase during the transition from TiO_2_(B) to anatase and the interface structure has been quite limited until now [25]. Because of the aspects explained above, in the present study, we report a detailed investigation on the effects of the concentration of NaOH on the structures, morphologies, and formation mechanisms of titanates obtained via hydrothermal synthesis. The anatase phase, obtained by the sonochemical method, was the starting material with average crystallite sizes of 5.2 ± 1.5 nm for the different concentrations of NaOH (from 1 to 10 M). Similarly, the temperature and reaction time were kept constant during the investigation (110 °C for 24 h). It was found that the morphologies of the titanate products were determined by the concentration of NaOH. Furthermore, during phase transformation, three processes were identified under hydrothermal conditions in alkaline solutions. These mechanisms are the self-assembly of nanofibers and nanosheets, the Ostwald ripening process, and the self-development of hollow TiO_2_ mesostructures. The set of these processes leads to the evolution from nano to mesoscale morphology.

## 2. Materials and Methods

### 2.1. TiO_2_-Anatase Synthesis of As-Prepared Sample by Sonochemical Procedure

The pure TiO_2_-anatase nanoparticles were prepared as follows: 30 mL of [(CH_3_)_2_ CHO]_4_ Ti (TTIP, Aldrich, St. Louis, MO, USA) as a titanium source was mixed with acetone (30 mL, reagent grade) and methanol (30 mL, reactive grade); then, this mixture was stirred for 15 min at room temperature to form a homogeneous suspension. After this time, the reaction mixture was exposed to ultrasound irradiation at 20 kHz for 50 min. After ultrasound treatment, the chemical solvents were evaporated using a magnetic mixer–heater plate at 60 °C up to the moment the remnant material was liquid-free and had a dry appearance [23]. Here, we denote these TiO_2_ nanoparticles from sonochemical synthesis as the as-prepared sample.

### 2.2. Hydrothermal Synthesis of TiO_2_ Heterostructures

**Figure 1:**
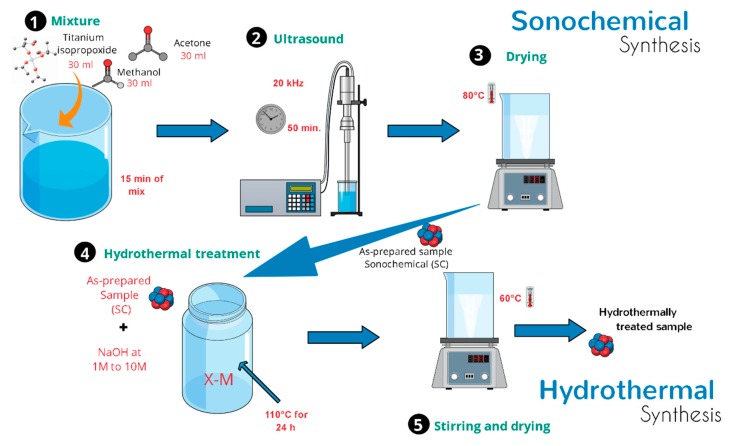
Schematic diagram of the synthesis process.

In a typical synthesis, 2.0 g of as-prepared TiO_2_-anatase sample was mixed in an aqueous sodium hydroxide (NaOH) solution at room temperature until forming a homogeneous suspension. The resulting mixture was simultaneously stirred using a magnetic stirrer. After a few minutes, a turbid white precipitation was formed. Then, the mixture was transferred into a 500 mL Teflon-lined autoclave reactor that was placed in an electric oven. The hydrothermal synthesis was conducted at 110 °C for 24 h. After the processing time, the mixture was allowed to naturally cool down to room temperature. Finally, white precipitates were collected by centrifugation and then washed several times with distilled water and ethanol. The same procedure was applied to different samples of TiO_2_-anatase using higher concentrations of NaOH in order to elucidate the evolution of TiO_2_ heterostructures. Throughout this work, a set of acronyms is used: XM, where X represents the concentration of NaOH, from 1 to 10 M. Figure 1 shows a schematic illustration of the process of sonochemical and hydrothermal synthesis.

### 2.3. Characterization

X-ray powder diffraction (XRD) patterns for the as-prepared sample of TiO_2_ and its hydrothermally treated samples were obtained using a Bruker diffractometer D8 Focus with monochromated high-intensity Cu Kα radiation (λ = 1.5406 Å) at 35 kV and 25 mA, and data were collected from 2θ = 5° to 2θ = 80° with a step of 0.20°. High-resolution transmission electron microscopy (HRTEM) images of TiO_2_ heterostructures were observed by an FEI-Titan (Hillsboro, OR, USA) operated at 300 kV (Cs = 1.2 nm). SEM images were obtained by a field emission scanning electron microscope (FESEM, FEI-Nova 200 Nanolab, Hillsboro, OR, USA) operated at 10 kV.

## 3. Results and Discussion

### 3.1. As-Prepared Sample Characterization

**Figure 2:**
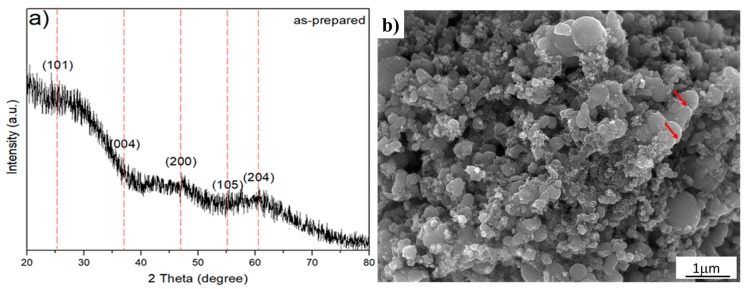
As-prepared sample. (**a**) Nanocrystalline TiO_2_ XRD pattern of the sample synthesized by ultrasonic radiation for 50 min, whereby the dashed lines correspond strong diffraction peaks according to JCPDS card No. 21-1272 (anatase TiO_2_); (**b**) Typical FESEM image depicting the cavitation effect by sonochemical means.

The XRD analysis of the as-prepared sample, TiO_2_-anatase synthesized sonochemically, yielded a plot intensity versus angle of diffraction as shown in Figure 2a. The XRD analysis shows an appreciable X-ray broadening and asymmetrical peaks with low intensity, characteristic of polycrystalline material with a nanometric crystallite size. The nanocrystallite size and lattice strain affect the Bragg peak in different ways. Both these effects increase the width and intensity and also shift the 2θ peak position in accordance with the results reported in the literature [26]. Additionally, the high background is associated with the crystallite size and lattice strain. Indeed, the lattice strain arises from crystal imperfections, such as lattice dislocation. Other sources of strain can include the grain boundary triple junction, contact or sinter stresses, and stacking faults [27]. The experimental XRD pattern was compared with the JCPDS card No. 21-1272 (anatase TiO_2_), and dashed lines were located at 2θ = 25.28, 48.05, and 62.69°, which are the strong diffraction peaks associated with the anatase phase. Moreover, a systematic shift of the diffraction peaks to smaller interplanar distance (*d*) values (or higher 2θ degrees) for the diffraction reflections can be observed. In order to elucidate and support the XRD analysis, as shown in Figure 2a, high-resolution electron microscopy was used to correlate the XRD analysis with the morphological and structural properties. Therefore, the morphology and crystalline structure information were analyzed. Thus, Figure 2b shows a typical SEM image of the sample synthesized by ultrasonic means. This figure shows that the ultrasonic irradiation of the liquid–powder suspension produces high-velocity interparticle collisions—neck formation zones denoted by red color arrows. Certainly, the formation of neck and grain boundaries is an interesting feature because the structure of these regions controls the transformation mechanisms, which minimizes the total surface energy by diffusional mass transport. Furthermore, the SEM micrograph also suggests that the influence of sonochemical means in the synthetized anatase is capable of generating changes in the surface morphology and reactivity [28,29]. Therefore, the as-prepared sample can strongly increase the surface area and mass transfer between two phases, both of which enhance and improve the diffusion coefficient on the interface mixing more than conventional agitation, including the rapid formation of TiO_2_ nanoparticles with narrow size distributions and high purities. However, the resultant collisions are capable of creating structural defects which, in turn, could explain the XRD spectrum of the as-prepared sample. For this reason, the XRD pattern of the as-prepared sample, shown in Figure 2a, is attributed to the crystallite size and structural defects produced by ultrasonic irradiation effects shown in Figure 2b. Consequently, we applied HRTEM analysis to support a deeper structural analysis. Furthermore, natural structural defects, such as grain boundaries between the particles generated by ultrasonic means, must be of considerable interest because this kind of crystalline defect could control diffusion rates and transformation mechanisms during the hydrothermal treatment.

**Figure 3:**
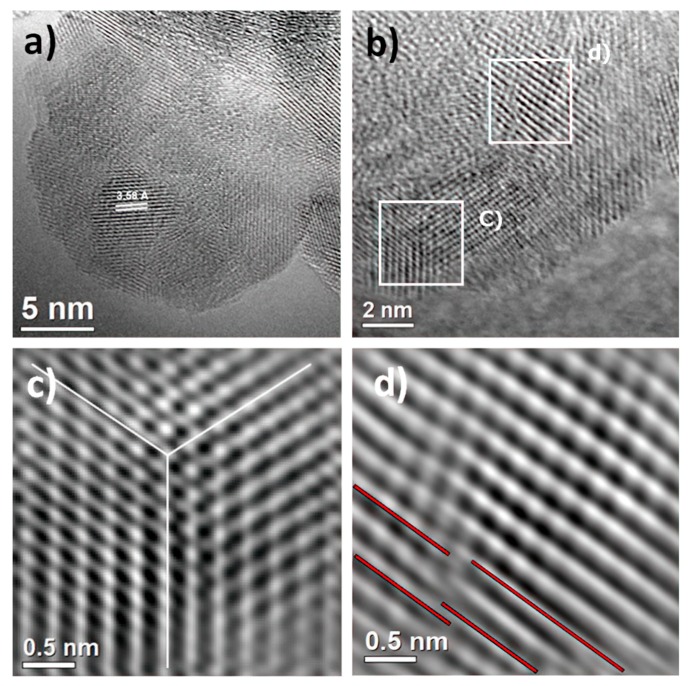
High-resolution transmission electron microscopy (HRTEM) of as-prepared sample. (**a**) Polycrystalline anatase phase with [101] projection; (**b**) the edge of the sample shows the formation of structural defects, indicated by white boxes, (**c**) such as twinning or homophases; (**d**) existence of dislocations in the sample.

To establish the correlation between the XRD pattern and FESEM analysis, electron irradiation and imaging of the as-prepared sample were carried out by HRTEM. These observations at atomic resolution are shown in Figure 3a–d. HRTEM analysis confirms anatase phase formation by sonochemistry. Also, the electron micrograph in Figure 3a shows the formation of crystalline material in nature with the domain in different orientations and, as a consequence, grain boundary formation. The average crystallite size was determined as 5.2 ± 1.5 nm, and the anatase phase was identified in the [101] projection. At the same time, it is necessary to remark that the measured interplanar spacing of the lattice plane was 3.58 Å instead of 3.52 Å. This result could be attributed to the presence of structural defects. In order to avoid any structural interference, such as the Moiré effect, the HRTEM micrograph was extended to the edge of the sample, shown in Figure 3b; in this micrograph, the existence of structural defects is evident, indicated by white boxes. These defects, such as twinning or homophase boundaries, are depicted in Figure 3c, and dislocation generation is presented in Figure 3d. This set of defects generates microstrains in the crystal structure. Thus, the formation of the nucleus and the growth of the nanocrystal is increased due to the direct influence of ultrasonic radiation. However, the reduction of particle sizes intrinsically generates structural defects that stem from the necessity of redistributing internal energy in the material and definitely the cavitation effect during the synthesis method. Therefore, high-resolution electron microscopy supports the evidence of the appreciable broadening and asymmetrical peak reflections in the XRD and FESEM analyses of the as-prepared sample.

### 3.2. Hydrothermal Process

#### 3.2.1. XRD Analysis

**Figure 4:**
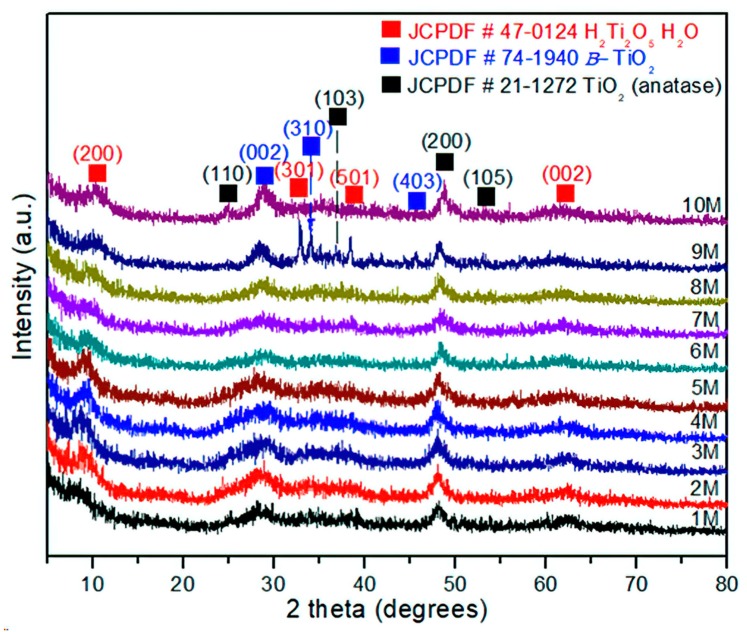
XRD of the TiO_2_-anatase samples treated with NaOH at concentrations of 1–10 M by the hydrothermal process.

The experimental XRD patterns of the products treated by hydrothermal process with NaOH from 1 to 10 M are displayed in Figure 4. The XRD analysis of the sample corresponding to 1 M NaOH shows a weak reflection at 2θ = 8.65°. The incoming experiments showed a constant shift and asymmetrical reflections, as can be seen in the sample treated with 10 M NaOH, 2θ = 10.52°. This sample was identified as H_2_Ti_2_O_5_∙H_2_O, (JCPDS 47-0124), corresponding to an orthorhombic crystalline structure and primitive Bravais lattice. These changes in the structural behavior could be considered as the influence of the hydrothermal treatment under alkaline conditions. Indeed, the layered formation of H_2_Ti_2_O_5_∙H_2_O instead of Na_2_Ti_9_O_19_ (JCPDS 33-1293, monoclinic structure) indicates major stability in the H_2_Ti_2_O_5_-H_2_O structure due to hydrogen bonding between layers [30]. Moreover, the surface corrosion generated by the hydrothermal conditions promotes structural defects, grain boundary saturation, and nanometric size. This result suggests that the introduction of impurities (atoms) in the anatase structure along with microstrains could explain the high background observed in the XRD spectra in Figure 4. Moreover, an interesting phenomenon can be observed in Figure 4, where the diffraction patterns and peak intensities are associated with plane (200), corresponding to the anatase phase and 1–9 M NaOH concentration, almost remaining constant at 2θ~48.12° and with 10 M NaOH at 2θ~48.32°. This fact is also significant because it points to the fact that once this crystalline plane is formed (200), it would not be involved in the structural changes, regardless of NaOH concentration, time, or temperature, remaining independent. In addition, it is possible to establish that this crystallographic plane reached its metastable thermodynamic equilibrium at a crystallite size of 7.5 ± 1.3 nm and remained steady under the experimental conditions reported. On the other hand, the crystallographic planes (101) of anatase and (002) of TiO_2_(B) have undergone several structural changes associated with continuous dissolution and recrystallization, until these planes reached their structural stability at experimental conditions of 10 M NaOH in accordance with the intensity and symmetric reflections. Finally, during the phase transformation or coexistence of both phases, the main crystallographic planes involved are (101) anatase and (002) TiO_2_(B), (JCPDS-74-1940). Hence, the phase transformation is characterized by a dissolution–recrystallization–redissolution process until the crystallite size reaches 12.7 ± 1.8 nm; then, the grain size has achieved metastable thermodynamic equilibrium in the system. These microstructural effects have not been reported in the literature until now.

#### 3.2.2. Field Emission Scanning Electron Microscopy Analysis (FESEM)

##### Surface Morphology

**Figure 5:**
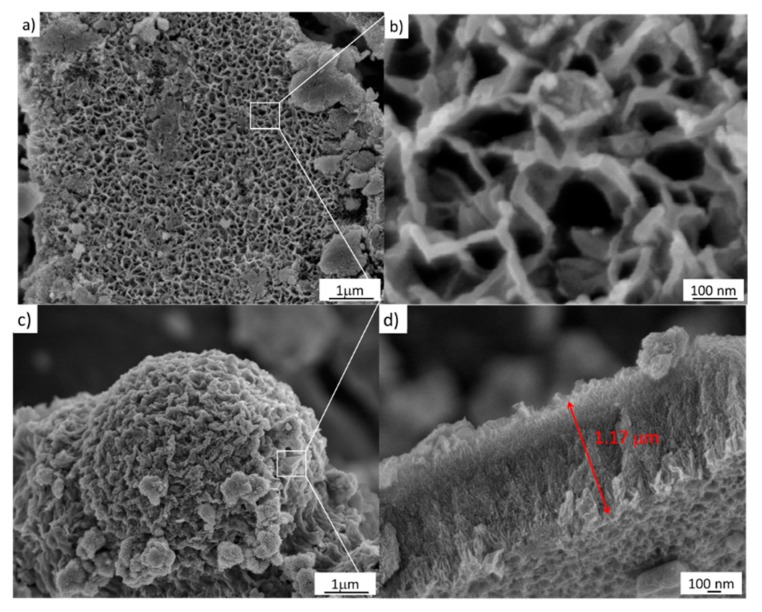
FESEM images of the samples treated hydrothermally at 110 °C for 24 h. (**a**,**b**) 1 M NaOH; (**c**,**d**) 2 M NaOH.

The FESEM image of the sample treated via a simple hydrothermal chemical process at 1 M NaOH is depicted in Figure 5a. Close packing can be clearly seen in this image at the large scale, and a hollow structure with a size around of 100 ± 30 nm is observed, as shown in Figure 5b. Furthermore, the sample also shows a mesoporous structure, also shown in Figure 5c. Moreover, Figure 5c shows the chemical corrosion that occurs using 2 M NaOH as well as a cross-section of the sample. As can be seen, this micrograph depicts oriented nanoarrays grown vertically with an average length of 1 µm, shown in Figure 5d.

**Figure 6:**
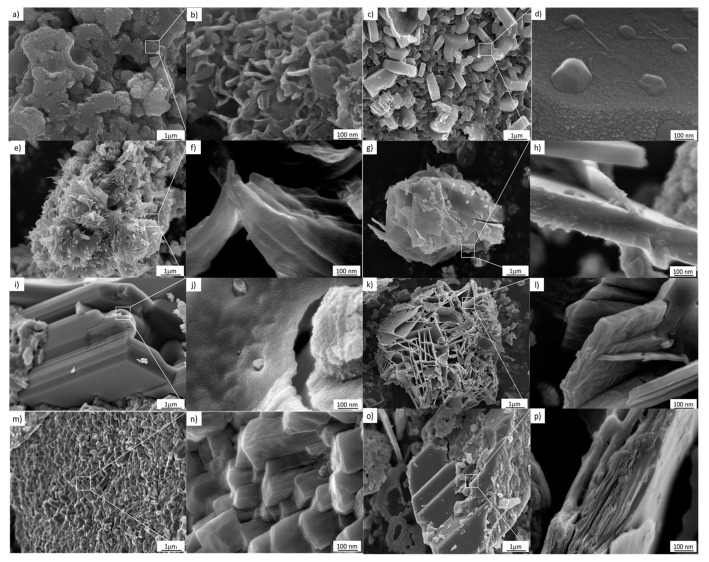
FESEM images of the samples treated hydrothermally at 110 °C for 24 h. (**a**,**b**) 3 M, (**c**,**d**) 4 M, (**e**,**f**) 5 M, (**g**,**h**) 6 M, (**i**,**j**) 7 M, (**k**,**l**) 8 M, (**m**,**n**) 9 M, (**o**,**p**) 10 M.

The FESEM images, shown in Figure 5, also demonstrate the effects of the NaOH concentration on the formation and transformation from anatase to titanate nanostructures in relation to Figure 4. Therefore, at this point, it is possible to establish that the stages that lead to phase transformation from TiO_2_-anatase to H_2_Ti_2_O_5_–H_2_O–TiO_2_(B) are subject to thermal effects in the as-prepared sample, 5.2 ± 1.5 nm, that involve nucleation, growth, and coarsening that stem from the redistribution of energy in the system. Subsequently, the thermal effects change the rate and outcome of the transformation, and could result in the appearance of an unusual state of phase; in particular, in nanosystems. This modification begins with a nucleation process and is accompanied by cluster growth. Here, the formation of anatase starts with a size of 4.2 ± 1.5 nm, and experiences eventual Ostwald ripening. This is a phenomenon observed in solid solutions that describes the change of an inhomogeneous structure over time; i.e., small crystals or sol particles dissolve, and redeposit onto larger crystals [23]. In this case, the hydrothermal process is considered, such as in a closed bath system, and the supersaturation drops as clusters nucleate and grow, causing an increase in the stable critical nucleus size. During the Ostwald ripening process, the subcritical clusters are considered to dissolve spontaneously, and so their mass is released to contribute to the further growth of the remaining clusters. This process is consistent with the analysis of FESEM images in Figure 6. 

As can be seen in Figure 6, the use of 3–10 M NaOH results in morphological changes in the TiO_2_ nanostructured anatase. This effect can be attributed to the evolution from a supersaturated phase to a single condensed ripened particle in equilibrium with the noncondensed phase. Therefore, a metastable supersaturated phase will not only generate nuclei; according to the classical theory of nucleation, it will also cause deposition on the nuclei and the consequent growth of clusters. In addition, according to the images in Figure 6a–p, the nucleation and the cluster growth processes will be present during the entire hydrothermal treatment procedure [24]. Furthermore, Figure 6a–p, corresponding to 7–10 M, indicates that as growth and nucleation rates decline, the Gibbs–Thompson effect becomes significant, meaning that smaller clusters dissolve and transfer their mass to large clusters, increasing their size. 

#### 3.2.3. High-Resolution Transmission Electron Microscopy Analysis

##### Crystallographic Analysis

**Figure 7:**
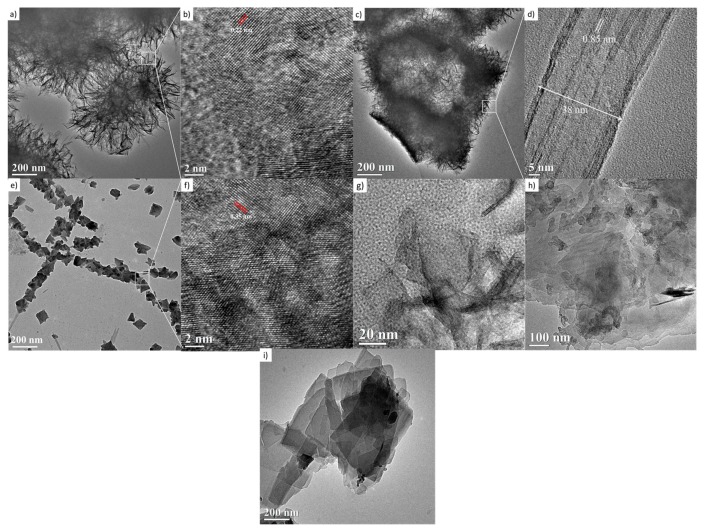
HRTEM images of the samples treated hydrothermally at 110 °C for 24 h. (**a**,**b**) 1 M; (**c**,**d**) 2 M; (**e**,**f**) 3 M; (**g**) 5 M; (**h**) 9 M; and (**i**) 10 M.

The TiO_2_-anatase samples treated hydrothermally with NaOH aqueous solution at 110 °C for 24 h at 1, 2, 3, 5, 9, and 10 M concentrations are shown in Figure 7. The HRTEM image in Figure 7a displays a cluster made up of large numbers of nanosheets, resembling mackerel scales, with a diameter of around 300 nm. The nanosheets are considered to have peeled off from the anatase during formation, and they grow during the hydrothermal treatment. The HRTEM image of nanosheets presents structural defects and non-uniform geometry. These two features are indicative of low crystallinity, consistent with the obtained XRD results. These nanosheets present an interplanar distance of about 0.22 nm, shown in Figure 7b, calculated after averaging measurements from several zones of this HRTEM image; it is necessary to state that this fringe distance does not correspond to the crystalline planes indexed in the XRD analysis. Moreover, it is plausible that clusters in the sample are a set of hydrated nanosheets which lose water under vacuum and when they are exposed to the 300 kV electron beam. Figure 7c contains granules composed of a layered structure with an interlayer distance of ca. 0.85 nm, shown in Figure 7d, which is close to the reported interlayer distance of the nanotubes [24]. 

**Figure 8:**
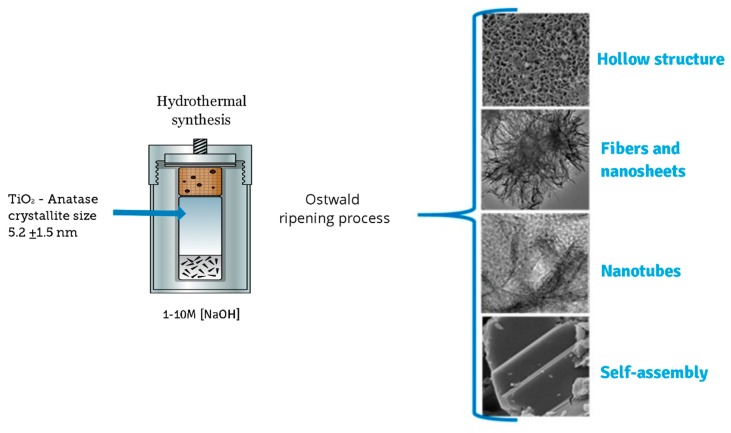
Sequential events for the synthesis of the different nanostructures in the given experimental conditions.

In this case, the layers could be peeled off with further acid washing, forming nanotubes. The image in Figure 7e,f shows a nanosheet with an interplanar distance of 0.35 nm, corresponding to the distance between the [101] planes of the anatase structure. The Figure 7g,h,i suggest the coexistence of nanotubes and nanosheets corresponding to 5, 9, and 10 M NaOH concentrations, respectively. The morphology and microstructure indicate the presence of nanosheets and nanotubes. The origin of the nanotube-like structures can be attributed to the rolling up of the sheet-like structures by surface forces [25]. It has been reported that the rolling up is a very fast event [31,32,33]. The formation of titanate nanotubes can be attributed to the hydration of anatase TiO_2_ through soft chemical interaction with NaOH. The rolling up of the titanate sheets is not a feasible process, and this condition is supported by the results obtained from FESEM analyses, where a path-dependent morphology was observed. Indeed, the results from HRTEM analyses are in good agreement with those obtained by XRD and FESEM analysis. Therefore, varying of NaOH concentration led to three distinct formation mechanisms. The reaction of TiO_2_ with NaOH yields the dissolved anatase phase and the temperature of 110 °C for 24 h promotes the nucleation and growth of nanosheets. The formation of these nanostructures is accelerated by increasing NaOH concentrations. We suggest that an alkali treatment environment breaks the bonds through hydroxyl bridging, leading to growth in the anatase [100] direction. This process along with lateral growth through oxo bridge formation in the [001] direction generates the crystalline sheets that then roll up to form nanotubes. These mechanisms could be attributed to three phenomena: the self-assembly of nanofibers and nanosheets, the Ostwald ripening process, and the self-development of hollow TiO_2_ mesostructures. The summary process described above is illustrated in Figure 8.

## 4. Conclusions

In summary, we report the sonochemical synthesis of a TiO_2_-anatase phase with a crystallite size of 5.2 ± 1.5 nm under different NaOH concentrations via hydrothermal reaction at 110 °C for 24 h. The concentration of NaOH affected the formation and transformation of titanate nanostructures mainly composed of H_2_Ti_2_O_5_-H_2_O. The formation of these nanostructures was accelerated by temperature effects and increasing NaOH concentration. The stages of formation and transformation of titanate nanostructures, nucleation, and growth are subject to hydrothermal conditions that stem from the redistribution of energy in the system. The obtained titanate nanostructures could be attributed to three phenomena: the self-assembly of nanofibers and nanosheets, the Ostwald ripening process, and the self-development of hollow TiO_2_ mesostructures.
